# COVID-19 outbreaks in care homes: How does size influence transmission dynamics? A cross-sectional study with implications for outbreak management in small care homes

**DOI:** 10.1017/S0950268825100757

**Published:** 2025-12-10

**Authors:** Catherine Carey, Éamonn O’Moore, Rita Huyton, Steve Willner, Anand Fernandes, Will Morton, Martyn Regan, Jackie Cassell

**Affiliations:** UK Health Security Agency, UK

**Keywords:** COVID-19, Care homes, Small care homes (SCHs), Transmission dynamics, Outbreak trajectories, Attack rate, Infection prevention, Public health policy

## Abstract

This cross-sectional study investigated how care home size influences COVID-19 transmission dynamics, focusing on outbreaks in England during the second wave of COVID-19 (Wave 2; December 2020 to March 2021) and the Omicron wave (December 2021 to February 2022). Using data from the UK Health Security Agency and the Care Quality Commission, positive SARS-CoV-2 test results were matched to care home registration and occupancy data, examining outbreak trajectories in homes of varying sizes and resident age groups. The study included over 90,000 positive cases across the two waves. Small care homes (SCHs, with 10 or fewer beds), predominantly housing younger adults, showed significantly higher early positivity rates: 42% of residents were positive at outbreak detection, rising to 61% by day 7. In contrast, larger homes had early positivity rates of only 3–6%. These findings suggest that SCHs, often designed for communal living, facilitate rapid within-home transmission similar to household settings. The study concludes that outbreak control strategies in SCHs should differ from those in larger care homes, emphasizing proportionate, individualized approaches that consider resident vulnerability and minimize disruption to social support systems. These results have broader implications for managing future infectious disease outbreaks and support the development of tailored guidance based on care home size and resident demographics.

## Introduction

During the COVID-19 pandemic, care home residents in England were disproportionately affected, experiencing high rates of severe illness and mortality [1]. Throughout the pandemic, older age, frailty, and underlying health conditions were consistently the main individual-level risk factors for severe COVID-19 outcomes, including hospitalization and death [2]. In 2021, the median age of residents in care homes for adults over 65 in England was 86 years and 5 months [3]. Prior to the rollout of the COVID-19 vaccination programme on 8 December 2020, and during the emergence of SARS-CoV-2 Variants of Concern (VOCs), the heightened vulnerability of older care home residents led to extensive and prolonged restrictions on visits, communal activities, and staff movement, on a scale not previously seen. Vulnerability to both severe COVID-19 outcomes and the unintended effects of public health and social measures [4] varied considerably between different types of care homes. Residents of care homes for younger adults primarily require care due to learning disabilities, autism, or other neurodevelopmental conditions. Although a proportion of these residents were at increased risk of severe outcomes due to underlying health conditions, many were not.

Care homes in England are registered with the Care Quality Commission (CQC) according to specialism, with distinct registration for caring for older adults over 65 years and younger adults under 65, and the option to register for more than one category of care [5]. Small care homes (SCHs), defined in line with CQC criteria as those registered with 10 or fewer beds, generally have communal facilities such as kitchens and living areas. They typically provide homes for residents under 65 years with learning and/or physical disabilities. These residents may actively engage in external leisure activities, education, training, and employment. From a transmission perspective, SCHs are often comparable to settings registered with the CQC as supported living or extra care housing and may differ only in that accommodation is contracted separately from care [6]. During the COVID-19 pandemic, supported living and extra care housing settings were advised to follow care home guidance whenever residents shared substantial communal facilities and most received CQC-regulated personal care [7], in contrast to settings providing only social support, such as cooking, cleaning, or shopping.

Mathematical modelling used to inform care home policy during the COVID-19 pandemic primarily focused on larger care homes with over 30 beds, overlooking SCHs with household-like characteristics. However, outbreaks in SCHs can be expected to show a high early attack rate due to dense social networks among a small number of residents. These residents often have minimal or no physical disability, unlike those typically living in larger care homes. As a result, a highly transmissible virus like SARS-CoV-2 would be expected to spread rapidly within an SCH, leading to high incidence in the first few days and limited opportunity for hidden or prolonged transmission chains.

In this study, we aimed to analyse COVID-19 outbreak trajectories and attack rates in relation to care home type with a view to informing evidence-based and differentiated approaches to future outbreaks.

## Methods

### Setting

The study aimed to include all care homes in England and their resident populations, during two key periods of the COVID-19 pandemic: Wave 2 (10 December 2020 to 1 March 2021) and the Omicron wave (15 December 2021 to 21 February 2022). These periods were chosen to capture the dynamics of COVID-19 transmission during two phases of the pandemic for which high-quality diagnostic data were available, with particular focus on the emergence of the highly transmissible Omicron variant.

### Study population

The analysis included care homes with two or more residents testing positive for SARS-CoV-2 within a 14-day period during the specified study windows. Residents were eligible if they had a positive SARS-CoV-2 test that could be linked to a CQC registered care home, enabling linkage to CQC registration data.

### Data sources

The UKHSA Epidemiology Cell (EpiCell) line list for positive COVID-19 tests [8] is a person-level dataset for laboratory-confirmed cases, derived from the Second-Generation Surveillance System (SGSS) and subsequently deduplicated and cleaned by EpiCell. SGSS is the UKHSA’s laboratory surveillance system for notifiable infectious diseases and antimicrobial resistance in England. In collaboration with the Geospatial Team, EpiCell then identified care homes via Unique Property Reference Number linkage. The resulting dataset includes each care home’s address, a specimen date, and date of death (if within 28 days of a positive test).

The CQC register [9] provides up-to-date information on active care home locations in England, including bed capacity and registration type. The Capacity Tracker [10] is a web-based application that enables care homes to share a core subset of data, including available capacity, in real time. Between May 2020 and 31 March 2022, submissions were incentivized via the Adult Social Care Infection Control and Testing Fund [11], and monthly reporting is now mandatory under the Health and Care Act 2022.

Care home size, registration type, and outbreak characteristics were ascertained using CQC-registered postcodes from the CQC Register for Active Locations [12] and matched to the UKHSA EpiCell line list for positive SARS-CoV-2 tests to perform descriptive analysis. Care home residents were identified using linkage to CQC data; homes designated for older people were those marked as serving ‘Older People’ and/or ‘dementia’ in the CQC register. All others were classified as homes for younger adults. While some younger adults may reside in homes registered for older people, the extent of misclassification was considered minimal.

### Bias

To avoid counting ongoing outbreaks, care homes with any cases in the 28 days prior to each study period (10 December 2020 to 1 March 2021 and 15 December 2021 to 21 February 2022) were excluded. Records marked as ‘Staff’ in the EpiCell dataset were also excluded. Cases that could not be linked to a CQC postcode and care homes with missing capacity data were also excluded.

Care home resident denominators for calculating transmission patterns over a 50-day period were derived from occupancy data in the Capacity Tracker. Care home occupancy was chosen as the most appropriate denominator for estimating relative transmission patterns between homes of different sizes because average care home occupancy in England is 80%. This approach minimized potential bias related to large care homes with many unoccupied beds. However, for comparison and to inform policymaking contexts where current occupancy may not be known, we also calculated transmission patterns using registered bed capacity.

### Study size

The sample size was determined based on the availability of data from care homes during the study periods. All care home residents who tested positive for COVID-19 between 10 December 2020 and 1 March 2021, or 15 December 2021 and 21 February 2022, and could be matched to a CQC-registered care home were included.

Exposure variables included care home size, defined by number of residents and categorized as small care homes (SCHs; ≤10 beds), mid-sized homes (11–49 beds), and large homes (≥50 beds), and registration type, distinguishing homes for older people from those for younger adults; separate analyses were conducted using bed count. Outcome variables comprised the attack rate, defined as the proportion of residents testing positive for SARS-CoV-2, at outbreak detection and subsequent time points, along with key outbreak characteristics.

### Statistical methods

Descriptive analyses were conducted to characterize outbreaks by care home size, using the proportion of residents positive for COVID-19 at outbreak detection and subsequent time points. No formal statistical comparisons were made. A sensitivity analysis was performed using registered bed numbers rather than resident counts.

## Results

### Demographics of the care home resident population

In March 2022, England had 15,134 CQC-registered care homes with 457,183 beds (Table 1). Of the 4,525 SCHs, 3,234 (71.5%) catered to younger adults and 1,291 (28.5%) to older adults, totalling 27,585 beds. Nearly half of all younger adult beds were in SCHs, compared with 2.1% of older adult beds.

**Table 1.** Care home type, size, and distribution by registration type, March 2022

Care home beds	Older adults care homes	Younger adults care homes	Total	% older adults	% younger adults
1–10	1,291	3,234	4,525	28.5	71.5
11–24	2,190	808	2,998	73.0	27.0
25–49	4,348	191	4,539	95.8	4.2
50–215	3,010	62	3,072	98.0	2.0
Total	10,839	4,295	15,134	71.6	28.4

*Note:* Percentages refer to the proportion of care homes in each size category.

Most care homes for older adults were mid-to-large-sized: 4,348 (40.1%) had 25–49 beds, and 3,010 (27.8%) had 50 or more beds, while only 62 (1.4%) of care homes for younger adults had 50 or more beds. These patterns reflect a structural divergence in care provision: younger adults are predominantly cared for in smaller homes, whereas older adults are largely cared for in mid-to-large-sized homes.

Table 2 shows resident demographics by home size and type. Across all homes, the median age of residents who tested positive for COVID-19 was 83 years (IQR 66–89). In SCHs, residents were younger, with a median age of 49 years (IQR 34–60). In mid-to-large care homes for older adults, median ages ranged from 86 to 87 years (IQR 79–91). These differences highlight the demographic heterogeneity of the care home sector and the varying needs of residents by age and home type.

**Table 2.** Distribution of care homes England by size, type, and median age, March 2022

CQC Registered beds	Older people					Younger people					Total beds				
	Median age	Age Q1	Age Q3	Registered beds	Residents	Median age	Age Q1	Age Q3	Registered beds	Residents	Median age	Age Q1	Age Q3	Registered beds	Residents
1–10	72	68	79	8,356	6,902	44	32	55	19,229	16,649	49	34	60	27,585	23,551
11–24	86	79	91	40,515	34,308	49	35	57	12,401	10,558	79	57	89	52,916	44,866
25–49	87	81	91	156,093	127,884	48	34	57	6,453	5,344	85	75	90	162,546	133,288
50–215	86	80	91	209,774	161,834	46	32	57	4,362	3,135	84	75	90	214,136	164,978
Total	86	80	91	414,738	330,928	45	31	56	42,445	35,686	83	66	89	457,183	366,683

*Note:* Median age and quartiles (Q1–Q3) of residents by care home size and type. Registered beds and residents are counts as of March 2022.

### Cases: Wave 2 and Omicron wave

During Wave 2 (10 December 2020–1 March 2021), 40,266 positive COVID-19 tests from the UKHSA EpiCell dataset were successfully matched to 5,655 unique CQC-registered care home postcodes. For the Omicron wave (15 December 2021–21 February 2022), 53,108 positive tests were matched to 6,973 care homes. An additional 316 care homes were excluded due to missing occupancy data in the Capacity Tracker, preventing accurate calculation of attack rates.

### COVID-19 attack rate: Omicron wave

**Figure 1.**
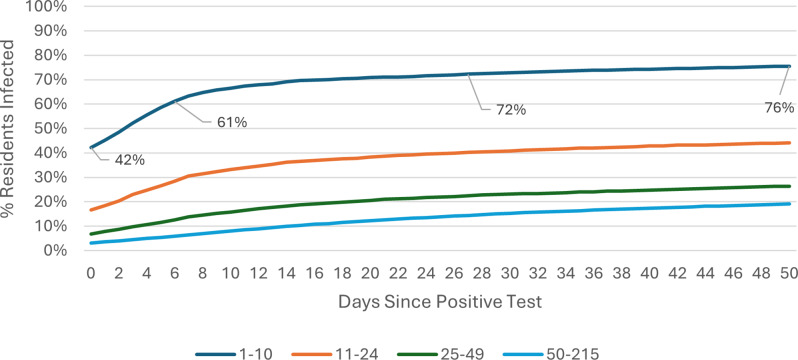
Outbreak trajectories during the Omicron wave in all care homes, by care home occupancy (15 December 2021–21 February 2022).

**Figure 2.**
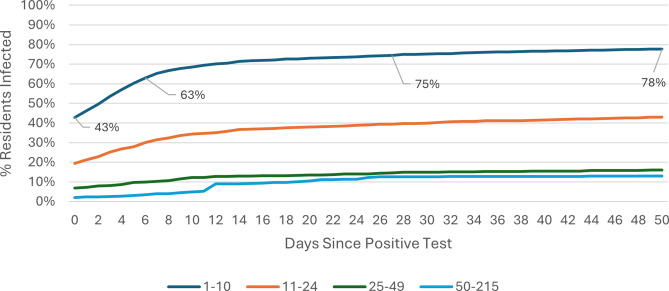
Outbreak trajectories during the Omicron wave in care homes for younger adults, by care home occupancy (15 December 2021–21 February 2022).

**Figure 3.**
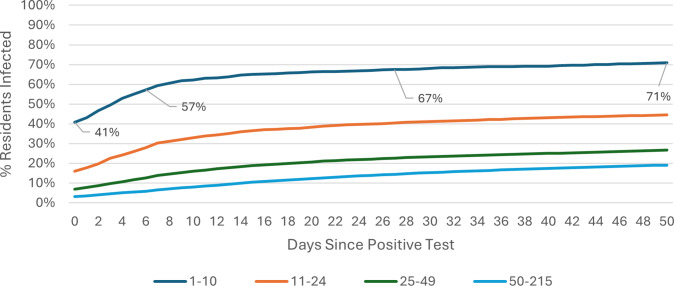
Outbreak trajectories during the Omicron wave in care homes for older adults, by care home occupancy (15 December 2021–21 February 2022).

The COVID-19 attack rate over the 50-day Omicron wave period was calculated using care home occupancy data as the denominator. Outbreak trajectories varied notably by care home size and registration type. Across all care homes (Figure 1), outbreaks in SCHs progressed rapidly, with 42% of residents already positive at the time of detection, rising to 61% by day 7, whereas care homes with 25–49 beds showed slower progression, increasing from 3% to 6% over the same period. In care homes for younger adults (Figure 2), SCHs showed a steeper trajectory, with positivity rising from 43% at day 0 to 63% by day 7. Among SCHs registered for older adults (Figure 3), progression was slightly slower, increasing from 41% to 57%, while in care homes for older adults with 50+ beds, positivity remained substantially lower, rising from 3% to 6% over the same timeframe.

Outbreak trajectories during Wave 2 followed a similar pattern but at lower levels prior to the emergence of the more transmissible Omicron variant [13]. These results are also shown in Supplementary Table 1, calculated using care home occupancy as the denominator. A parallel analysis for both Wave 2 and Omicron, using CQC-registered bed numbers as the denominator, is presented in Supplementary Table 2.

## Discussion

The transmission dynamics of COVID-19 outbreaks in SCHs of all types differed markedly from patterns seen in larger care homes. SCHs consistently showed high attack rates at first detection and by day 7, reaching a plateau earlier than larger care homes. Overall attack rates by the end of the outbreak were also higher in SCHs, particularly in homes for younger adults. This marked difference has important implications for policymakers, informing which outbreak measures are likely to be effective in controlling transmission according to care home size.

Beyond care home size, infection prevention and control (IPC) measures and other non-pharmaceutical interventions (NPIs) likely influenced outbreak patterns. CQC’s *COVID-19 Insight* reports in 2020 [14] indicated generally strong assurance around IPC across home types, although implementation and intensity varied. Staff were required to wear masks, and cohorting was commonly used to limit transmission. However, adherence may have been more challenging in smaller care homes for younger adults due to communal living, social interaction needs, and, in some cases, cognitive or behavioural factors.

These contextual challenges were compounded by differences in staffing structures. Larger care homes typically employ staff with formal IPC training and dedicated infection control roles but experience higher staff movement between homes, a recognized transmission risk. In contrast, SCHs, particularly those for younger adults, rely on small, multi-role teams, sometimes including live-in staff who provide both personal and domestic support, supporting continuity of care but often with limited formal IPC training. At the start of the COVID-19 pandemic, national data on IPC training levels and compliance in England were lacking. While basic IPC training was mandatory for personal care providers, it was not included in the skills framework for learning disability care, where staff frequently work flexibly across home and community environments [15]. Taken together, these structural, behavioural, and staffing factors likely underpinned the rapid outbreak progression observed in SCHs.

### Relationship to previous literature

The COVID-19 pandemic generated extensive research on outbreaks in care homes for older adults. One of the largest studies, VIVALDI [16], followed residents and staff across care homes in England with a median resident age of 86, bed capacity of 40–63, and occupancy of 82.5%. The study found that two doses of COVID-19 vaccine were 61.7% effective against infection and 96.4% effective against death during the first year. Although immunity waned over time, a booster largely restored protection; by the Omicron wave, effectiveness against infection had declined due to both waning and immune escape, while protection against severe outcomes remained largely preserved.

Vaccine rollout initially prioritized older adult residents in larger care homes, ensuring early access to first and second doses during the winter of 2020–2021. In contrast, residents of SCHs for younger adults were vaccinated later and generally had lower uptake. This delayed and less comprehensive immunity contributed to rapid outbreak progression in SCHs, highlighting the need for proportionate, tailored outbreak control measures that balance infection prevention with resident wellbeing.

Subsequent studies reinforce the protective effect of vaccination in care homes. Giddings et al. [17] examined outbreak severity and duration across English care homes between November 2020 and June 2021, reporting that following COVID-19 vaccine introduction, outbreak duration and resident attack rates were substantially reduced, with typical attack rates in larger homes ranging from 13% to 27% by day 28. These population-level benefits complement the individual-level vaccine effectiveness reported by the VIVALDI study [16], which found that vaccination markedly reduced infection, hospitalization, and death among care home residents and lowered infection risk among staff, contributing to reduced transmission within care homes. Earlier investigations, including Ladhani et al. [18], focused on larger London care homes with 43–100 residents in April 2020, reporting resident attack rates of 70–75% before vaccination and widespread outbreak control measures were implemented.

An international review of respiratory outbreaks in elderly care homes [19] similarly found high attack rates, often exceeding 50%, and prolonged transmission in large facilities during seasonal pathogen circulation. These findings contrast with the lower overall attack rates observed in larger English care homes in our study (13–27%) and underscore the distinctive outbreak dynamics of smaller homes, where rapid within-home transmission led to early plateaus. Outbreaks of this type are underrepresented in published literature, which tend to describe prolonged or complex events in larger care homes. Our study helps to address this evidence gap by providing national-level data on SCH outbreak trajectories to inform proportionate, tailored infection-control strategies.

### Strengths and limitations

In interpreting our findings, it is important to note that COVID-19 outbreaks in care homes for older adults typically had multiple genomic lineages, indicating multiple introductions of the virus from the community [20]. We did not have genomic data on these outbreaks; this is important context both for interpreting our study and informing policy decisions.

Our study used a complete national dataset for England, prospectively collected during the COVID-19 pandemic. COVID-19 test results were matched to CQC-registered care homes using postcodes, excluding approximately 15% of homes as only those with unique postcodes could be linked. It is unclear whether this disproportionately affected smaller or larger care homes. This missingness is likely Missing Not At Random (MNAR), since exclusion probability is related to underlying characteristics; for example, larger care homes often share postal addresses, particularly when multiple facilities are co-located. Such systematic missingness cannot be fully addressed using standard statistical methods, as it is associated with unobserved structural factors [21].

We also lacked data on resident health status, environmental factors, social and community activities, and new admissions. The absence of these variables may contribute to unmeasured sources of bias, further compounding the challenges posed by MNAR data.

### Implications for clinical practice, policy, and future research

Guidance should consider the implications of swift early transmission and faster progression to eventual outbreak size, which occurs in SCHs, noting the wider evidence cited above on multiple introductions in larger care homes. SCHs for younger adults, by design, emulate shared households, with communal facilities that may facilitate rapid resident-to-resident transmission.

Given these patterns and the limited scope for hidden chains of COVID-19 transmission, our data provide empirical evidence to support the management of SCHs in a similar way to households in the wider community. This will require strategies to protect particularly vulnerable residents from cases of active infection during outbreaks of COVID-19, including supporting residents to participate in protective measures.

During the COVID-19 pandemic, some settings other than care homes (e.g., supported living, extra care settings) were managed in the same way as homes of any size. Importantly, given the strikingly different patterns of transmission in smaller vs. larger care homes, future outbreak management should consider the likelihood that prolonged control measures in SCHs are unlikely to reduce transmission or eventual outbreak size. This distinction is crucial for assessing trade-offs for active younger populations in care homes, whose wellbeing may be substantially impacted by the loss of external visits for leisure, education, and other family or community activities. This was recognized in December 2022 guidance published by the Department of Health and Social Care DHSC [22] to provide care homes with autonomy to initiate COVID-19 outbreak management risk assessments to inform decisions about which outbreak measures best fit their individual settings. Testing guidance was streamlined for SCHs to reduce the number of tests staff and residents needed to take in the event of an outbreak.

While specific to COVID-19, these findings may have implications for differential management of other infectious hazards in care homes, including periods of closure to admissions and visiting. This should be explored in future research and evaluation. Despite the limitations in this dataset, the opportunity to analyse patterns of transmission and outbreak trajectories across all care homes highlights the value of timely, accessible, and systematically collected data to inform tailored advice in future outbreaks. A comprehensive observatory enabling characterization by care home size and type could strengthen national preparedness for future infectious hazards while supporting evidence-based interventions that protect residents and safeguard their quality of life.

## Supporting information

10.1017/S0950268825100757.sm001 Carey et al. supplementary material 1 Carey et al. supplementary material

10.1017/S0950268825100757.sm002 Carey et al. supplementary material 2 Carey et al. supplementary material

## Data Availability

A limited dataset analysed during the current study is available from the corresponding author on reasonable request, subject to data protection.

## Supplementary material

The supplementary material for this article can be found at http://doi.org/10.1017/S0950268825100757.

## References

[1] Chief Medical Officer, Chief Scientific Adviser. (2022) Technical report on the COVID-19 pandemic in the UK. In: Department of Health and Social Care, editor. A technical report for future UK chief medical officers, government chief scientific advisers, National Medical Directors and public health leaders in a pandemic. Chapter 8.2: Care homes. London: His Majesty’s Government.

[2] Hollinghurst J, et al. (2022) COVID-19 risk factors among 14,786 care home residents: An observational longitudinal analysis including daily community positive test rates of COVID-19, hospital stays and vaccination status in Wales (UK) between 1 September 2020 and 1 May 2021. Age and Ageing 51(5). 10.1093/ageing/afac084 PMC9070807 35511729

[3] Office for National Statistics. Older people living in care homes in 2021 and changes since 2011. Office for National Statistics (2021) https://www.ons.gov.uk/peoplepopulationandcommunity/birthsdeathsandmarriages/ageing/articles/olderpeoplelivingincarehomesin2021andchangessince2011/2023-10-09 (accessed 7 April 2025).

[4] World Health Organization (2025) A decision framework for effective, equitable and context-specific public health and social measures during public health emergencies: Decision Navigator. Geneva: WHO https://www.who.int/publications/i/item/9789240108611 (accessed 23 September 2025).

[5] Care Quality Commission (2022) Registration under the Health and Social Care Act 2008. The Scope of Registration. London: Care Quality Commission.

[6] Care Quality Commission (2015) Guidance on Regulated Activities for Providers of Supported Living and Extra Care Housing. London: Care Quality Commission.

[7] Care Quality Commission (2025) Glossary of Terms. London: Care Quality Commission https://www.cqc.org.uk/about-us/glossary-terms (accessed 23 September 2025).

[8] UK Health Security Agency (2022) UKHSA Epidemiology Cell (EpiCell) Line List for Positive COVID-19 Tests. London: UKHSA.

[9] Care Quality Commission (2022) Using CQC Data. London: Care Quality Commission. https://www.cqc.org.uk/about-us/transparency/using-cqc-data (accessed 10 September 2025).

[10] NHS England, Department of Health and Social Care (2022) Capacity Tracker reports: Utilisation by region and location https://capacitytracker.com/reports/utilisation-report/by-region-and-location (accessed 10 September 2025).

[11] Department of Health and Social Care (2021) Adult Social Care Infection Control and Testing Fund (ICTF). GOV.UK https://www.gov.uk/government/publications/adult-social-care-infection-control-fund (accessed 10 September 2025).

[12] Care Quality Commission (2022) CQC Directory Register for Active Locations. London: Care Quality Commission. https://drive.google.com/drive/folders/0B1jvn_rdpdEzMUtiNVoyeW9rb2M?resourcekey=0-J1nm1TwV6Vf_N9DArEe6XQ (accessed 10 September 2025).

[13] Bálint G, Vörös-Horváth B and Széchenyi A (2022) Omicron: Increased transmissibility and decreased pathogenicity. Signal Transduction and Targeted Therapy 7, 151. 35525870 10.1038/s41392-022-01009-8 PMC9077027

[14] Care Quality Commission (2020) COVID-19 insight: Infection prevention and control. London: Care Quality Commission. https://www.cqc.org.uk/sites/default/files/20200916_covidinsight_issue04.pdf (accessed 10 September 2025).

[15] Skills for Health, Health Education England, NHS England (2019) Core capabilities framework for supporting people with a learning disability. London: Skills for Health. https://www.skillsforhealth.org.uk/wp-content/uploads/2020/11/Learning-Disability-Framework-Oct-2019.pdf (accessed 10 September 2025).

[16] Shrotri M, et al. (2022) Duration of vaccine effectiveness against SARS-CoV-2 infection, hospitalisation, and death in residents and staff of long-term care facilities in England (VIVALDI): A prospective cohort study. The Lancet Healthy Longevity 3, e470–e480. 10.1016/S2666-7568(22)00147-7. 35813279 PMC9252508

[17] Giddings R, et al. (2021) Changes in COVID-19 outbreak severity and duration in long-term care facilities following vaccine introduction, England, November 2020 to June 2021. Euro Surveillance 26(46). 10.2807/1560-7917.ES.2021.26.46.2100995 PMC8603404 34794537

[18] Ladhani SN, et al. (2020) Investigation of SARS-CoV-2 outbreaks in six care homes in London, April 2020. EClinicalMedicine 26, 100533. 32923993 10.1016/j.eclinm.2020.100533 PMC7480335

[19] Utsumi M, et al. (2010) Types of infectious outbreaks and their impact in elderly care facilities: A review of the literature. Age and Ageing 39, 299–305. 20332371 10.1093/ageing/afq029

[20] Tang S, et al. (2021) Mass testing after a single suspected or confirmed case of COVID-19 in London care homes, April-May 2020: Implications for policy and practice. Age and Ageing 50(3), 649–656. 10.1093/ageing/afab054. 33620453 PMC7929429

[21] Dong Y and Peng CYJ (2013) Principled missing data methods for researchers. Springerplus 2, 222. 10.1186/2193-1801-2-222. 23853744 PMC3701793

[22] Department of Health and Social Care (2022) Changes to the universal use of face masks in adult social care for COVID-19 and COVID-19 outbreak management in care homes. London: DHSC.https://careprovideralliance.org.uk/assets/pdfs/dhsc-letter-on-use-of-face-masks-and-covid-outbreak-management-changes-15-dec-2022.pdf (accessed 10 September 2025).

[23] Health Research Authority (2013) Is my study research? London: HRA. https://www.hra-decisiontools.org.uk/research/ (accessed 10 September 2025).

