# Supplementary material for: COVID-19 outbreaks in care homes: How does size influence transmission dynamics? A cross-sectional study with implications for outbreak management in small care homes
Source: Epidemiol Infect. 2025 Dec 10;154:e8. doi: 10.1017/S0950268825100757 (PMC12813716; doi:10.1017/S0950268825100757)
Supplement: Carey et al. supplementary material 1 — Carey et al. supplementary material [file S0950268825100757sup001.docx]

|  | **Supplementary Table 1.** COVID-19 attack rates by wave and care home type, with resident numbers defined using Capacity Tracker occupancy. | | | | | | | | | | | | | | | | | | |  |  |  |  |
| --- | --- | --- | --- | --- | --- | --- | --- | --- | --- | --- | --- | --- | --- | --- | --- | --- | --- | --- | --- | --- | --- | --- | --- |
|  |  | | | | | | | | | | | | | | | | | |  |  |  |  |  |
|  | Care home registered beds | Younger adults care homes | | | | |  | Older adults care homes | | | | |  | All care homes | | | | |  |  |  |  |  |
|  |  | Number of cases (N) | Day 0 (%) | Day 7 (%) | Day 28 (%) | Day 50 (%) |  | Number of cases (N) | Day 0 (%) | Day 7 (%) | Day 28 (%) | Day 50 (%) |  | Number of cases (N) | Day 0 (%) | Day 7 (%) | Day 28 (%) | Day 50 (%) |  |  |  |  |  |
|  | Omicron Period 15 December 2021 - 21 February 2022 | | | | | | | | | | | | | | | | | |  |  |  |  |  |
|  | 1-10 | 3687 | 43 | 63 | 75 | 78 |  | 1601 | 41 | 57 | 67 | 71 |  | 5288 | 42 | 61 | 72 | 76 |  |  |  |  |  |
|  | 11-24 | 1714 | 19 | 30 | 39 | 43 |  | 6726 | 16 | 28 | 40 | 44 |  | 8440 | 17 | 28 | 40 | 44 |  |  |  |  |  |
|  | 25-49 | 270 | 7 | 10 | 15 | 16 |  | 20009 | 7 | 13 | 23 | 27 |  | 20279 | 7 | 13 | 22 | 26 |  |  |  |  |  |
|  | 50-215 | 108 | 2 | 3 | 13 | 13 |  | 18993 | 3 | 6 | 14 | 19 |  | 19101 | 3 | 6 | 14 | 19 |  |  |  |  |  |
|  | Wave 2 10 December 2020 - 01 March 2021 | | | | | | | | | | | | | | | | | |  |  |  |  |  |
|  | 1-10 | 3541 | 43 | 63 | 74 | 77 |  | 1511 | 41 | 57 | 68 | 70 |  | 5052 | 42 | 61 | 72 | 75 |  |  |  |  |  |
|  | 11-24 | 1521 | 19 | 29 | 37 | 40 |  | 5804 | 16 | 28 | 40 | 43 |  | 7325 | 17 | 28 | 39 | 42 |  |  |  |  |  |
|  | 25-49 | 274 | 7 | 10 | 15 | 16 |  | 14625 | 7 | 13 | 22 | 25 |  | 14899 | 7 | 13 | 22 | 25 |  |  |  |  |  |
|  | 50-215 | 54 | 2 | 4 | 9 | 10 |  | 12936 | 3 | 7 | 16 | 20 |  | 12990 | 3 | 6 | 16 | 20 |  |  |  |  |  |
|  | *Percentages represent the cumulative proportion of cases at each time point (Day 0, Day 7, Day 28, Day 50) from the start of the outbreak. | | | | | | | | | | | | | | | | | |  |  |  |  |  |
