# Supplementary material for: COVID-19 outbreaks in care homes: How does size influence transmission dynamics? A cross-sectional study with implications for outbreak management in small care homes
Source: Epidemiol Infect. 2025 Dec 10;154:e8. doi: 10.1017/S0950268825100757 (PMC12813716; doi:10.1017/S0950268825100757)
Supplement: Carey et al. supplementary material 2 — Carey et al. supplementary material [file S0950268825100757sup002.docx]

**Supplementary Table 2**. COVID-19 attack rates by wave and care home type, with resident numbers defined using CQC-registered beds.

| Care home registered beds | Younger adults care homes | | | | |  | Older adults care homes | | | | |  | All care homes | | | | |
| --- | --- | --- | --- | --- | --- | --- | --- | --- | --- | --- | --- | --- | --- | --- | --- | --- | --- |
|  | Number of cases (N) | Day 0 (%) | Day 7 (%) | Day 28 (%) | Day 50 (%) |  | Number of cases (N) | Day 0 (%) | Day 7 (%) | Day 28 (%) | Day 50 (%) |  | Number of cases (N) | Day 0 (%) | Day 7 (%) | Day 28 (%) | Day 50 (%) |
| OMICRON PERIOD 15 December 2021 – 21 February 20221 | | | | | | | | | | | | | | | | | |
| 1-10 | 3687 | 38 | 56 | 66 | 69 |  | 1601 | 35 | 50 | 59 | 62 |  | 5288 | 37 | 54 | 64 | 67 |
| 11-24 | 1714 | 17 | 26 | 34 | 37 |  | 6726 | 14 | 24 | 34 | 38 |  | 8440 | 14 | 24 | 34 | 38 |
| 25-49 | 270 | 6 | 8 | 12 | 13 |  | 20009 | 6 | 10 | 18 | 22 |  | 20279 | 6 | 10 | 18 | 21 |
| 50-215 | 108 | 2 | 3 | 10 | 10 |  | 18993 | 2 | 4 | 11 | 15 |  | 19101 | 2 | 4 | 11 | 15 |
| Wave 2 10 December 2020 – 01 March 2021 | | | | | | | | | | | | | | | | | |
| 1-10 | 3541 | 39 | 56 | 66 | 69 |  | 1511 | 35 | 50 | 58 | 61 |  | 5052 | 38 | 54 | 63 | 66 |
| 11-24 | 1521 | 16 | 24 | 32 | 34 |  | 5804 | 14 | 24 | 34 | 37 |  | 7325 | 14 | 24 | 33 | 36 |
| 25-49 | 274 | 6 | 9 | 12 | 14 |  | 14625 | 6 | 10 | 18 | 20 |  | 14899 | 6 | 10 | 18 | 20 |
| 50-215 | 54 | 2 | 3 | 7 | 7 |  | 12936 | 2 | 5 | 12 | 15 |  | 12990 | 2 | 5 | 12 | 15 |

*Percentages represent the cumulative proportion of cases at each time point (Day 0, Day 7, Day 28, Day 50) from the start of the outbreak.
